# Weight reduction among females undergoing laparoscopic ventral hernia repair: The role of Calcium and Vitamin D3

**DOI:** 10.12669/pjms.40.5.9047

**Published:** 2024

**Authors:** Saleh Abdulrahman AlMulhim, Mounther Mohammed AlNaim, Abdul Sattar Khan, Abdul Qadeer Memon, Haytham Mohammed AlArfaj, Ahmad A. Al Abdulqader, Abdulrahman Saleh AlMulhim

**Affiliations:** 1 Saleh Abdulrahman AlMulhim, MBBS. Family Medicine Physician, Department of Family Medicine, National Guard Hospital (NGHA), AlAhsa, Saudi Arabia; 2 Mounther Mohammed AlNaim, MBBS, SBFM, ABFM. Associate Consultant in Family Medicine, Department of Family Medicine, National Guard Hospital (NGHA), AlAhsa, Saudi Arabia; 3 Abdul Sattar Khan, Assistant Professor of Family & Community Medicine, Department of Family & Community Medicine, King Faisal University College of Medicine; 4 Abdul Qadeer Memon, FCPS, FICS. Assistant Professor of Surgery, Department of Surgery, King Faisal University College of Medicine, AlAhsa, Saudi Arabia; 5 Haytham Mohammed AlArfaj, Assistance Professor of Surgery, Department of Surgery, King Faisal University College of Medicine, AlAhsa, Saudi Arabia; 6 Ahmad A. Al Abdulqader, Assistance Professor of Pediatric Surgery, Department of Surgery, King Faisal University College of Medicine, AlAhsa, Saudi Arabia; 7 Abdulrahman Saleh AlMulhim, FRCSI, FICS, FACS. Professor of Surgery, Department of Surgery, King Faisal University College of Medicine, AlAhsa, Saudi Arabia

**Keywords:** Vitamin-D, BMI, Ventral Hernia Repair, Calcium Supplements

## Abstract

**Objective:**

To evaluate the role of Vitamin-D and calcium supplementation on preoperative weight reduction in obese women before laparoscopic ventral hernia repair.

**Methods:**

This double-blind clinical trial was conducted at the affiliated health centers of King Faisal University, Al-Ahsa, Saudi Arabia from January 2021 to December 2021. It included forty-five obese women aged 24-56 years, with body mass index (BMI) of 34.0–48.0kg/m^2^. They were randomly allocated into two groups; the Group-A (N=22) included obese women who received supplementation of 5000IU cholecalciferol (Vitamin-D_3_), and 1000mg calcium daily for 12 months, while the Group-B (N=23) received no treatment. Measurement of change in weight and BMI and comparison of their pre-operative weight reduction, laparoscopic operative time, and length of hospital stay was done.

**Results:**

There were no differences in patients’ biographic data between the two groups. During the study, Vitamin-D level in the patients increased and there was a significant positive association with weight loss. In group-A, the mean weight loss was 11.8±3.5 kg. At the end of first year, their BMI decreased from 36.1±1.6kg/m^2^ at baseline to 29.7±2.6 kg/m^2^, whereas in-group-B, the mean weight loss was 6.8±3.1 kg and their BMI decreased from 36.9±2.69kg/m^2^ at baseline to 32.7±0.93kg/m^2^. The operation time and the length of hospital stay were shorter in group-A (107 vs.128.min) and (3 vs. 5 days) respectively as compared to Group-B.

**Conclusion:**

Vitamin-D and calcium supplementation contributes to a remarkable weight reduction of preoperative obese female patients, which in turn is associated with significantly better outcome of laparoscopic repair of ventral hernia.

## INTRODUCTION

Obesity is a main health problem worldwide; it is an epidemic disease.^1^,^2^ It is associated with diabetes mellitus, other endocrine dysfunctions^3^,^4^ and surgical disorders like abdominal wall hernias. Abdominal wall hernia is common in obese patients; nevertheless, obesity is a risk factor for the repair failure^5^ and other postoperative fatal complications.^6^ Therefore, reducing the patients’ weight before the procedure is one of the essential requirements to minimize the complications of the laparoscopic hernia repair.

Several studies have linked obesity to low calcium intake, Vitamin-D insufficiency, and detected an inverse correlation between serum level of 25-hydroxy vitamin-D {25(OH) D} and body weight.^7^-^9^ Likewise, there is sufficient evidence regarding the role of increasing intake of calcium, and Vitamin-D in body weight control, indicating that Vitamin-D potentiates weight loss.^10^-^13^ This is the first study to our knowledge that was performed in obese women before laparoscopic ventral hernia repair in whom we investigated the role of cholecalciferol (Vitamin-D) and calcium supplementation on weight reduction before the surgery.

## METHODS

This double-blind clinical intervention-controlled trial was conducted from January 2021 to December 2021 and followed-up for 12 months. All the female patients above 18 years’ age, having abdominal hernia (umbilical, para-umbilical) with BMI ≥ 30 kg/m^2^ registered at primary health care clinics in AlAhsa, Saudi Arabia region and referred to surgical outpatients’ clinic were invited to participate. Out of total 206 obese Saudi women, only 45 consented to participate in the study.

During the study period, all participants were advised to practice low-calorie intake diet with regular visits to nutrition clinic, combined with regular exercise. They were also advised to report directly to the hospital if they experienced any adverse symptoms, and closely monitor to detect abnormal laboratory results like hypervitaminosis and hypercalcemia in Group-A. Every subject underwent laparoscopic umbilical hernia repair after finishing the 12 months period.

### Randomization

Family physicians at the primary health centers (PHCs) in the region assigned the patient to either experimental or controlled groups randomly, 50% in each group. The researcher generated an unpredictable random allocation sequence and concealed it until the decision had been taken for giving calcium and Vitamin-D supplementation.^14^ The Group-A (experimental) included 22 obese women (mean body weight 96.3 ±11.4 kg; BMI 36.7±4.2 kg/m2; mean age 41 ±5.8 years) who were provided with 5,000 IU oral cholecalciferol (Vitamin-D3) and 1,000 mg calcium citrate taken daily after breakfast. While Group-B (control) included 23 obese women (mean body weight 95.6±12.5 kg; BMI 36.9±3.9 kg/m2; mean age 42±4.6 years) who were provided by no supplements.

### Procedure

At PHCs, all the patients underwent through a routine medical history, physical examination, laboratory, and radiological investigations. All the patients then were evaluated by surgical and family care consultants. In addition, some more information had been collected including age, BMI, ASA status, associated co-morbidities, type of hernia and previous operations. Their serum calcium, Vitamin-D (25(OH) D), PTH, liver and renal functions were measured as baseline and every two months as well as the weight and BMI. The cutoff used for Vitamin-D levels were as follows: deficient (<50 nmol/L), insufficient (50-75 nmol/L), and desirable (>75 nmol/L).^15^

### Statistical Analysis

The mean and standard deviation have been calculated for the descriptive analysis after feeding data in SPSS version 23. The p-value 0.05 was considered as significant. Comparison of the changes in the mean values of the weight and Vitamin-D level was done by applying student-unpaired t-test, while their comparison at baseline and after 12 months values was conducted by independent paired t-test.

### Ethical consideration

This study was approved by the medical ethics review committee of the King Faisal University (Research number: 2020-12–47, dated 27/12/2020). An informed consent had been taken from the patients for participation in the study and explained them all the consequences of participating in the study. It was totally voluntary participation and participants had full right to refuse anytime in between the study period. The participants were assured of the confidentiality of the data.

## RESULTS

No significant difference found in mean age (p=0.997), weight (p=0.603), BMI (p=0.232), Vitamin-D (p=0.549), co-morbidity (p=0.999) and types of hernia (p=0.712) in baseline characteristics observed between Group-A and B. However, there is significant difference in the meantime of operation (106.7±2.45 minutes) and mean hospital stays (2.77± 0.97 days) in both the groups (Table-I). At baseline in Group-A, their means are as follows: serum 25-OH-D = 45.09 (±0.97), age = 41.27 (±9.20) years, weight = 95.95 (±1.32) kg and BMI = 36.18 (±1.62). Similarly, in group B, the serum 25-OH-D is = 45.52 (±3.20), age = 41.26 (±9.28) years, weight = 96.50 (±4.98) kg and BMI = 36.99 (±2.69) (Table-1). Out of total 45 patients, 43(95.6%) were Vitamin-D deficient i.e. <50 nmol/L; 2(4.4%) had Vitamin-D insufficiency i.e. 50-74.9 nmol/L. Comparison among weight, BMI and Vitamin-D during the study period is shown in Table-II.

**Table-I:** Biographic and baseline data of the patients (N=45).

Variables	Group-A (Experimental)	Group B (Control)	P – value
Patients No (%)	22 (48.88%)	23(51.11%)
Age (mean ± SD)	41.27±9.20	41.26±9.28	0.997
Weight (mean ± SD)	95.95±1.32	96.50±4.98	0.603
BMI (mean ± SD)	36.18±1.62	36.99±2.69	0.232
Serum 25 (OH)-D (mean ± SD)	45.09±0.97	45.52±3.20	0.549
Time of operation (mean ± SD)	106.7±2.45	127.74±6.08	0.000
Hospital Stay in days (mean ± SD)	2.77± 0.97	05±1.68	0.000
***Co-Morbidity No. (%)***			
No diseases	12 (54.5%)	13(56.6%)	0.999
Diabetes	05 (22.7%)	05(21.7%)	
Hypertension	03 (13.6%)	03 (13%)	
Others	02 (9.1%)	02 (8.7%)	
***Types of hernia***			
Umbilical	10 (45.5%)	07(30.5%)	0.712
Para-umbilical	09 (40.9%)	11(47.8%)	
Incisional	2 (9.1%)	04(17.4%)	
Recurrent	1 (4.5%)	01(4.3%)	

**Table-II:** Comparison in Body Weight, BMI and Vitamin-D during the Study.

	Group-A (Experimental)			Group B (Control)		

Periods	BMI kg/m2 Mean± SD	Body Wt. – kg Mean± SD	Vitamin-D nmol/L Mean± SD	BMI kg/m2 Mean± SD	Body Wt. – kg Mean± SD	Vitamin-D nmol/L Mean± SD
Baseline	36.1±1.6	95.9±1.3	45±0.9	36.9±2.6	96.5±4.9	45.5±3.2
After 2 months	36.2±5.3	94.3± 9.6	52±2.1	36.7±4.2	95.1± 9.7	46±2.5
After 4 months	35.4±3.7	91.7± 8.5	59±3.2	36.1±5.8	94.7 ± 5.3	47±1.3
After 6 months	33.6±5.1	89.6± 6.9	66±3.4	35.7±3.7	93.5±7.7	49±3.0
After 8 months	31.8±6.2	87.4 ±5.7	75±1.1	34.5±3.6	92.1 ±2.3	52 ±1.0
After 10 months	30.4±2.4	85.4 ±3.8	80±0.9	33.5±2.9	90.6 ±4.1	55±2.1
After 12 months	29.7±2.6	84.1± 5.6	89.7±5.0	32.7±0.9	89.7 ±7.8	56.6±1.5

Table-II also shows comparison of weight reduction in Group-A & B during the study. In Group-A, the mean weight loss was 11.8-±3.5 kg. Their BMI decreased from 36.1±1.6kg/m^2^ at baseline to 29.7±2.6 kg/m^2^ at the end of the first year. During the first two months period, there was significant change in serum 25(OH) D (p=0.000), however, neither the weight (p=0.532) nor BMI (p=0.735) were changed as compared to baseline. Later, there was a gradual increase in serum 25(OH) D until the end of the study. The significant improvement in the serum level of 25(OH) D (p=0.000) as well as the weight loss (p=0.000) happened from months six to the end of the year. Serum parathyroid hormone (PTH) decreased gradually until the end, whereas serum calcium corrected initially, and remained unchanged over the study period. The mean length of stay was three days for Group-A. The mean operative time in Group-A was 106.7±2.45 minutes.

Whereas in Group-B, the mean weight loss was 6.8±3.1 kg. Their BMI decreased from 36.9±2.69kg/m^2^ at baseline to 32.7±0.93kg/m^2^ after the first year. Serum 25-(OH)-D3, PTH, and Ca levels did not change much in this group. During the first two months, there was no significant change in serum 25(OH)D (p=0.558), however, neither the weight (p=0.540) nor BMI (p=0.848) were changed as compared to baseline. There was an improvement in the serum level of 25(OH) D (p=0.000), however, the weight loss (p=0.122) occurred from baseline to months six and at the end of the year was significant (p=0.001). All the patients after 12 months’ time underwent laparoscopic hernia repair under general anesthesia. In the group B, the mean operative time was 127.74±6.08 minutes and the mean length of stay was five days. Table-II and Table-III show comparison between the two groups after 12 months on bi-monthly basis, their standard deviation (SD) and standard error of mean (SEM).

**Table-III:** Comparison of mean values of baseline and after 12-months data of BMI, Body weight & Vitamin-D level in an intervention Group-A

Pairs	Paired Differences Mean	SD	SEM	95% CI		P-Value

				Lower	Upper	
Body Mass Index (Baseline) vs Body Mass Index (After 12 months)	-11.80909	5.88	1.253	-14.416	-9.201	.000
Body Weight (Baseline) vs Body Weight (After 12 months)	- 6.45000	2.35	0.503	-7.49	-5.403	.000
Vitamin-D Level (Baseline) vs Vitamin-D Level (After 12 months)	44.636	5.27	1.125	42.29	46.97	.000

## DISCUSSION

**Fig.1:**
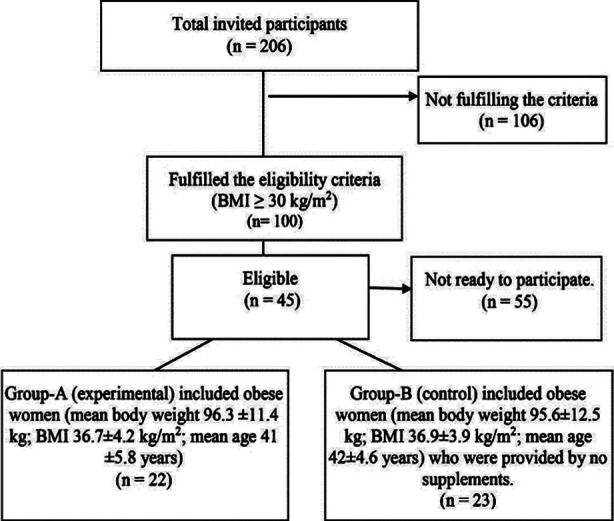
Randomization of the participants.

During the first two months, there was no significant change in serum 25(OH) D. Hence, neither the weight nor BMI changed significantly. Later, there was gradual increase in serum 25(OH) D until the end of the study. The significant improvement in its serum level as well as the weight loss occurred from months six to the end of the year (Fig.1). Serum PTH decreases gradually, whereas serum calcium corrected initially, and remained unchanged over the study period.

**Fig.2:**
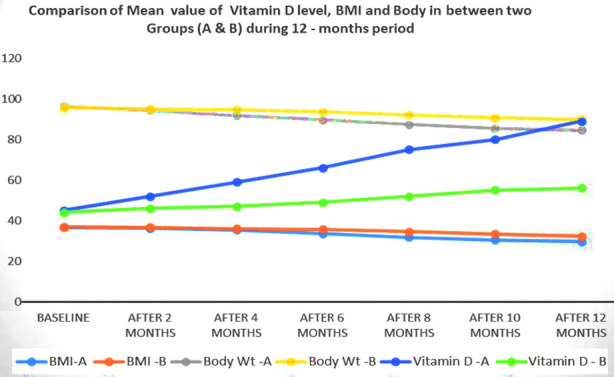
Comparison of Mean value of Vitamin-D level, BMI and Body in between two Groups (A & B) during 12 - month’s period.

Low Vitamin-D status is a common global disorder, with overweight and obesity often coexist in persons with Vitamin-D (25[OH] D) deficiency and low intakes of calcium. Many studies have demonstrated that calcium intake is inversely associated with body weight, and Vitamin-D intake is related to lower adiposity.^16^,^17^ There is considerable attention on the possible mechanisms by which these nutrients regulate body weight/BMI.^18^,^19^

The relationship of the Vitamin-D deficiency with obesity is poorly understood. The studies conducted in this regard suggest multifactorial mechanism. The possibility includes lower dietary intake, reduced cutaneous synthesis, reduced intestinal absorption and altered metabolism but all these are unproven. Other explanations state that there is impairment of 25-hydroxylation and 1-α hydroxylation processes due to decreased expression of the 1-α hydroxylase in obese people. It hinders the activation of the Vitamin-D. Vitamin-D is rapidly deposited in adipose tissue and slowly released in the blood. Increased surface area may cause the volumetric dilution of all the concentrates in blood including Vitamin-D. Hence, the weight reduction decreases the volume of adipose tissue which in turn decreases the body surface area and deposition of Vitamin-D in it, releasing it into the blood.^20^

The recommended daily allowance (RDA) of Vitamin-D is 600 IU/day for adults. Overweight and obese individuals need higher doses. The meta-analysis study of Lotito et al indicates that 1000-4000IU Vitamin-D supplementation needed in the overweight and obese population.^18^ Therefore, in this work we provided our patients with 5,000 IU oral cholecalciferol (Vitamin-D3) and 1,000 mg calcium citrate taken daily after breakfast. Our results were in concurrence with many studies^21^ reported the crucial effect of Vitamin-D and calcium supplementation on body weight. On the other hand, many other studies show no effect of supplementation on weight loss.^22^,^23^

All herniorrhaphies in both groups were started and completed laparoscopically by one senior laparoscopic surgery consultant. We noticed that mean operative time and mean hospital stay are shorter in patients received supplementation. This can be related to some extent to weight loss in Group-A, as operations in the obese are associated with technical problems such as difficulty with access, retraction of the abdominal wall, mobilization of the viscera and reduced working space due to intra-abdominal fat.

This is perhaps the first study which presents the outcomes of Vitamin-D3 and calcium supplementation on weight reduction in obese female patients before laparoscopic ventral hernia repair for preventing the postoperative complications.

### Limitations of the study

The sample size was relatively small, which limits the statistical power to detect smaller differences between the groups. Furthermore, the follow-up period of the patients in this study was short. Longer-term follow-up could provide more information about the outcome.

## CONCLUSION

It is suggested that Vitamin-D and calcium supplementation contributes to a remarkable reduction in weight of preoperative obese female patients, which in turn is associated with significantly better outcome of laparoscopic repair of ventral hernia.

### Authors’ Contribution:

**AlMSA:** Collected the data and wrote the manuscript.

**AlNMM:** Reviewed the data and wrote the manuscript.

**KAS:** Reviewed and edited the manuscript.

**MAQ:** Wrote, reviewed and edited the manuscript.

**AlHM** and **AlAA:** Wrote the manuscript.

**AlAS**: Reviewed, edited and approved the manuscript. He is also responsible for the integrity and accuracy of the study.
